# Discriminating circular polarization of light: Left or right?

**DOI:** 10.1038/s41377-024-01694-w

**Published:** 2025-01-03

**Authors:** Wanhee Lee, Changsoon Cho

**Affiliations:** 1 Department of Material Science and Engineering, Pohang University of Science and Technology (POSTECH), Pohang, Republic of Korea; 2 Institute for Convergence Research and Education in Advanced Technology, Yonsei University, Seoul, Republic of Korea

**Keywords:** Optical sensors, Metamaterials

## Abstract

Achiral dielectric nanostructures provide an efficient method for discriminating left- and right-circularly polarized photons, leveraging the photothermoelectric effect.

## Graphical abstract

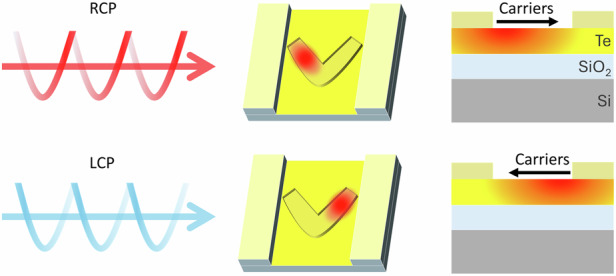

Circular polarization occurs when an electromagnetic wave comprises two orthogonal linear electric field components with a π/2 phase shift. This results in an electromagnetic field that rotates circularly, creating left-circularly polarized (LCP) and right-circularly polarized (RCP) light, as shown in Fig. 1^1^. CPL discrimination has applications in quantum optics^2^, chiral molecule analysis^3^, and remote sensing^4^. In quantum encryption, for example, CPL can encode data in photon polarization states, requiring efficient CPL detectors for secure photonic applications.

**Fig. 1:**
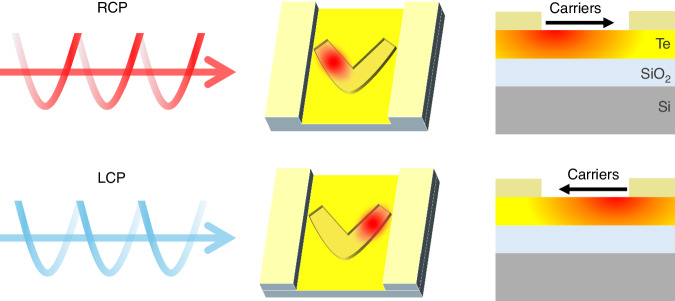
Photothermoelectric effect inducing opposite charge carrier flows under circularly polarized illuminations

Typical CPL detectors employ external optical filters that selectively transmit photons of specific polarization. LCP and RCP detection is achieved by utilizing two detectors with complementary filters. The polarization selectivity of these detectors is quantified by the discrimination ratio (DR):

1 $${\rm{DR}}=\frac{2\left|\left({R}_{\rm{LCP}}-{R}_{\rm{RCP}}\right)\right|}{\left({R}_{\rm{LCP}}+{R}_{\rm{RCP}}\right)}$$

where *R*_LCP_ and *R*_RCP_ indicate the photoresponses to LCP and RCP, respectively. DR increases when *R*_LCP_ ≫ *R*_RCP_ or *R*_LCP_ ≪ *R*_RCP_, reaching 2 when either LCP or RCP is perfectly blocked by filters. To realize such a polarization selectivity, previous studies have investigated various optical systems based on organic semiconductors^5^, hybrid perovskites^6^, chiral structures^7^, chiral nanoparticles^8^, and topological insulators^9^, exhibiting high DRs of >1 in the visible spectrum.

In this context, Zhang et al.’s recent work, published in *Light: Science & Applications*, brings an interesting approach to CPL discrimination^10^. Zhang et al. demonstrate that DR can exceed previous limits by designing a system where *R*_LCP_ and *R*_RCP_ have opposing signs. When *R*_LCP_ = −*R*_RCP_, DR theoretically becomes infinite, providing unparalleled discrimination efficiency. Their device, composed of V-grooves and paired stripe electrodes on a Te nanosheet (Fig. 1), generates photovoltage with reversed polarity for LCP vs RCP illumination.

Zhang et al.’s design relies on achiral dielectric nanostructures, diverging from traditional chiral-based designs. Although chiral geometries are commonly used in CPL discrimination, recent findings suggest that even achiral plasmonic metasurfaces can distinguish LCP from RCP light by generating different near-field modes^11,12^. Upon LCP or RCP illumination, the field localizes on one side of the V-groove, inducing a temperature gradient and charge carrier flow through the photothermoelectric effect. Consequently, a photovoltage with opposite polarity arises for LCP and RCP light, making the achiral structure ideal for high DR due to symmetric temperature profiles (*R*_LCP_ ~ −*R*_RCP_).

Figure 2 illustrates ideal photovoltage (*V*_ph_) responses under illumination with various quarter-wave plate (QWP) angles. RCP and LCP light correspond to QWP angles of *N* × π and (*N* + 0.5) × π, respectively. Linear electrode arrays without nanostructures produce cosine-shaped photovoltage responses, with a period of π/2. As the period matches the angle difference between RCP and LCP, the circular polarization is not distinguishable, while the structure can be better suited for detecting linear polarizations^13^. On the other hand, with well-designed achiral nanostructures, such as V-grooves, the response period increases to π. This results in opposite photovoltage signs for RCP and LCP light, enabling efficient discrimination of circular polarization. Zhang et al.’s experiments confirmed that their CPL detectors operate effectively across the visible spectrum, achieving high DRs: 107 at 405 nm, 20 at 520 nm, and 32 at 638 nm.

**Fig. 2:**
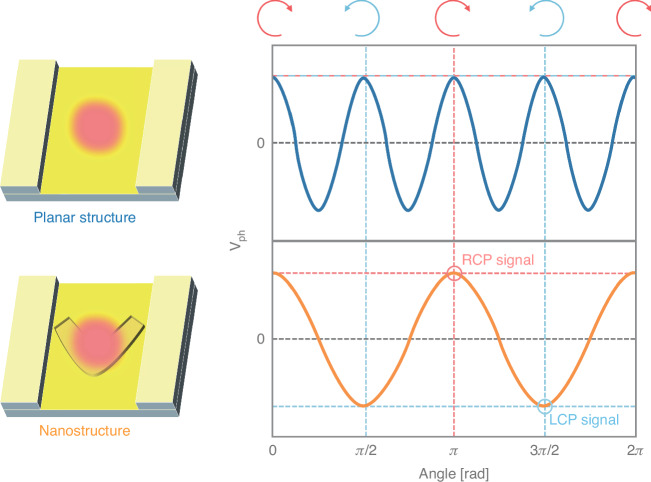
Ideal photovoltage (*V*_ph_) responses of planar and nanostructured photodetectors based on the photothermoelectric effect, ignoring various signal offsets and side effects

Although the proposed concept achieves high DRs, it is not intended to completely replace conventional CPL detectors but rather to complement them for different applications. While the device efficiently determines the direction of CPLs through the sign of *V*_ph_, it does not directly measure signal intensity. The magnitude of *V*_ph_ can increase due to either higher intensity or stronger polarization of the illumination, making it difficult to distinguish between these factors without an additional component to sense intensity. Furthermore, minimizing offset voltage will be essential for reliable operation under low-light conditions. Thus, this concept is most suitable for applications requiring immediate determination of CPL direction, such as binary optical communications, where direction matters more than signal magnitude or composition.
